# Breaking bad news in assisted reproductive technology: a proposal for guidelines

**DOI:** 10.1186/s12978-017-0350-1

**Published:** 2017-07-20

**Authors:** Daniela Leone, Julia Menichetti, Lorenzo Barusi, Elisabetta Chelo, Mauro Costa, Luciana De Lauretis, Anna Pia Ferraretti, Claudia Livi, Arne Luehwink, Giovanna Tomasi, Elena Vegni

**Affiliations:** 1 Department of Health Sciences, Università degli Studi di Milano, San Paolo University Hospital, Via di Rudinì 8, 20142 Milan, Italy; 2 Department of Psychology, Catholic University of Milan, Milan, Italy; 3 Assisted Reproductive Unit, Parma University Hospital, Parma, Italy; 4 Demetra Assisted Reproductive Center, Florence, Italy; 5 Ospedale Evangelico Internazionale, Assisted Reproductive Unit, Genoa, Italy; 6 Istituto Clinico Città Studi, Assisted Reproductive Center, Milan, Italy; 7 SISMER, Assisted Reproductive Center, Bologna, Italy; 8 Azienda Provinciale per i Servizi Sanitari- Provincia Autonoma di Trento, Assisted Reproductive Unit, Arco, Italy; 9 CRA, Assisted Reproductive center, Catania, Italy

**Keywords:** Art, Bad news, Communication, Buckman protocol, Infertility, Qualitative method, Spikes

## Abstract

**Background:**

The issue of breaking bad news in assisted reproductive technology (ART) has been only partially explored by literature, and although some recommendations are available, specific guidelines are lacking. The present study aimed to explore the applicability of the oncologic SPIKES Protocol to the ART context.

**Methods:**

Thirteen ART clinicians (7 gynecologists; 4 psychologists; 1 biologist; 1 obstetrician) completed the Critical Incidents Report (CIR) to describe the experience of delivering bad news in ART. The CIRs were first discussed with clinicians, then a focus group was created composed of 13 clinicians, one health communication expert and a patient to discuss the applicability of the six-step (SPIKES) Buckman Protocol to ART. The discussion was audiotaped, transcribed and analyzed with content analysis.

**Results:**

The SPIKES Protocol seems to fit ART consultations and participants found it practical and easy to understand. Some specificities were found for the ART context: the reiteration of bad news, the “patient” as a couple and the fact that ability to conceive is closely related to self-esteem, as well as to social and family identity. During the discussion of the SPIKES Protocol, participants highlighted the importance of: 1) providing a caring setting, by adding a reflection on the value of communication by phone; 2) exploring patients’ perceptions but also misinformation; 3) exploring patients’ desires and expectations, while balancing the need to be honest and clear; 4) applying Buckman’s suggestions for delivering information, and integrating clinical aspects with psychosocial ones; 5) managing and legitimizing patients’ emotions, in particular anger; 6) having a strategy for follow-up and supporting couples to make meaning of the ART experience.

**Conclusion:**

The proposal of a shared protocol for giving bad news in ART could be the starting point for training and experimental studies.

## Plain English summary

Breaking bad news in an assisted reproductive context can be a frequent occurrence due to low rates of success. Clinicians are often unprepared to manage this kind of communication, as literature on assisted reproductive technology (ART) lacks specific guidelines for managing difficult conversations, unlike in oncology where the six-step (SPIKES) Buckman Protocol was developed. The present study aimed to explore the applicability of the SPIKES Protocol to the ART context through a focus group of ART experts (7 gynecologists; 4 psychologists; 1 biologist; 1 obstetrician). First of all, participants completed the Critical Incidents Report (CIR) to describe the experience of delivering bad news. Thereafter, a focus group with ART experts together with an expert in health communication and a patient was conducted. Group discussion of CIRs was the starting point of the focus group, followed by discussion about the applicability of the SPIKES Protocol to ART. The discussion was audio-taped, transcribed and analyzed with qualitative content analysis.

This study found that the SPIKES Protocol fit ART consultations, even if the definition of bad news was found to be more controversial than in oncology, due to the fact that the ability to conceive was closely related to personal identity. The discussion of Buckman’s six-steps pointed out some specificities of the ART context, such as: telephone communication in the setting; the necessity to balance patients’ expectations with the need to be honest; the importance to integrate clinical aspects with psychosocial ones; the need to manage patients’ anger; the importance to help couples accept the clinical situation.

## Background

Having a child is a natural part of life for most couples; however, 9–15% of couples have problems conceiving [1]. Although great progress has been made in the treatment of infertility, success rates of assisted reproductive technologies (ART) are still only around 30% per cycle [2]. The inability to have a child, despite treatment, may cause psychological distress, depression and anxiety to effected couples [3–8]. Infertility treatments can be physically and emotionally demanding [9]. In the ART context communicating bad news to couples can be a frequent occurrence: the infertility diagnosis, the repeated failures in the treatment, and the clinical ineffectiveness of medical treatments are all bad news that professionals need often to communicate.

Disclosing difficult information is a complex process, and professionals are often unprepared to manage these communications [10], which may delay breaking bad news or its inappropriate disclosure. Poor communication between ART professionals and patients is commonly reported in the literature [11, 12]. Consequences of bad communication between patients and healthcare professionals include patients’ poor satisfaction with care, lower treatment compliance, reduced quality of care, and increased medical malpractice suits [13–18].

Helping healthcare professionals improve their communicational skills - especially in difficult situations, becomes crucial to avoid risks of negative patient-staff interactions [10]. Nevertheless, literature focuses principally on the psychological and emotional impact of infertility and treatment failure couples face [3–6], while it pays less attention on how clinicians can manage delivering bad news in the ART context. Some [10, 19] have highlighted the necessity for infertility specialists to be prepared in disclosing bad news. However, little research has been conducted on doctor-patient communication in medically assisted reproduction. Moreover, the ART literature lacks specific guidelines for managing difficult conversations.

It has been demonstrated that improved communication skills and strategies can be learned and retained long-term by health care providers [20–22]. An effort has been made to develop protocols and frameworks to assist healthcare professionals manage difficult conversations [22–25]. Among these, the Six-Steps (SPIKES) Buckman Protocol has been widely adopted and studied, particularly in the oncological setting [22, 23]. The SPIKES Protocol has been applied to different clinical settings and situations [26, 27]. Oncologists have found the SPIKES Protocol practical, easy to understand and useful in responding to the patient’s emotional reactions [22]. Moreover, it has been found that having a strategy to break bad news can increase physician confidence in disclosing unfavorable medical information [21, 28]. The evaluation of the SPIKES Protocol from the patient’s point of view is still unclear, but some studies indicate that it may not meet their needs [29, 30]. The aim of the present study was to explore the applicability of the SPIKES Protocol to disclosing bad news in the ART context.

## Methods

### Participants

Participants included both experts in ART and one ART patient. Experts were recruited among a larger group of ART professionals during a meeting on medical communication and through a snowball process (i.e., initial participants were asked to invite other potentially interested participants). Twelve-fifteen participants were purposively targeted in order to collect data from professionals from different disciplines and clinical contexts, in order to make group discussion rich and deep. The purposive sample included 13 ART professionals (7 gynecologists; 4 psychologists; 1 biologist; 1 obstetrician), 1 expert in health communication and 1 ART patient. The patient and the 13 ART professionals came from 8 private and public ART centers in Italy, the expert in health communication came from a University hospital in Milan. Each participant was informed about confidentiality and privacy procedures, the right to refuse participation at any point, and the audiotaping of the session. All participants gave informed written consent. They were asked to keep any personal information discussed in the focus groups confidential. All data that could have identified participants were removed from the transcripts to guarantee anonymity.

### Data collection

Participants were invited to join a half day group session, which included:

1. Individual completion of the Critical Incidents Report (CIR) [31, 32]. All the 13 ART professionals were asked to complete one CIR in order to bring out the participants’ experience and facilitate group discussion. The CIR was a written interview in which ART experts were asked to report a critical event in delivering bad news. Questions included impressions related to the event, learning outcomes, and factors that helped professionals in managing the event (Table 1). After discussing the CIRs a synthesis of what emerged from participants’ clinical experience was used to facilitate the focus group that followed.
2. A 4-h focus group [33, 34] was conducted to discuss the applicability of the SPIKES Protocol in the ART context. Two psychologists with expertise in health communication, facilitated the focus group. The interview guide for the focus group featured: 1) individual CIR and the participants’ experiences of delivering bad news in the ART setting; 2) how “bad news” is defined in the ART setting; 3) the applicability of the SPIKES protocol for breaking bad news in the ART setting (for every step of the SPIKES Protocol, participants were asked to discuss the applicability to their context and similarities/differences with the oncological setting). Insights on how to best adapt the protocol to the ART context were also collected. The focus group, carried out in Italian, was audio recorded and transcribed verbatim. The quotes selected for this article were translated by the researchers involved in the data collection, and later analyzed together with an Italian and English speaker. Finally, a native English speaker and professional translator was engaged to confirm translations [35]. A synthesis of the SPIKES Protocol [22, 23] is presented in Table 2.

**Table 1:** The Critical Incidents Report Guide

*1. Please describe a situation that left you unsatisfied in which you delivered bad news (or in which you assisted someone else delivering bad news) in your clinical practice.* *Please, describe the situation, actors involved, how, where, and when it happened, and why it was unsatisfactory in your opinion.*
*2. What meaning did you give to this critical event? Please, describe your impressions and feelings regarding the situation.*
*3. What did you learn from this experience? If you could go back, would you have acted differently?*
*4. Are there any tools, information, personal or professional skills of yours capable of helping you manage such event in a better way?*

**Table 2:** The Six-Step Buckman Protocol (SPIKES) (Adapted from [22, 23])

STEP	DESCRIPTION
Setting up	Be prepared for bad news conversations: find a private space, introduce oneself, involve significant others, sit down, manage interruptions.
Patient Perception	Assess the patient’s perception of the situation and what he/she already knows and wants to know in order to tailor the bad news communication to the patient’s level, correct any misinformation and determine the patient’s understanding and/or denial.
Invitation	Assess how much information the patient wants to know and seek the patient’s willingness before sharing. If patients do not want to know the details, offer to be available and answer any questions as they may arise in the future. Determine the bare minimum of information that is necessary to share and begin by focusing on that.
Knowledge	Before informing the patient, signal the patient that bad news is about to be conveyed. When sharing information, avoid medical jargon and excessive bluntness; provide information in small chunks and periodically check the patient’s understanding; repeat information several times.
Emotions	Respond to the patient’s emotional responses (shock, disbelief, anger and/or grief). Let the patient express his or her feelings; offer support by naming the patient’s emotions and normalizing such feelings. Use empathic response also to acknowledge the clinician’s own emotions.
Strategy and Summary	Summarize the main points and, if the patients are ready, discuss the treatments options available or follow-up plans. Frame the information and future hope in terms of what it is most meaningful to the patient and still possible to accomplish.

### Data analysis

Data was analyzed through a thematic analysis [36], in order to detect patterned responses and meanings on the topic of bad news in the ART setting and on the steps of the SPIKES Protocol. A hybrid approach of inductive (derived from the theoretical framework) and deductive (themes emerging from participants’ discussion) coding [37] was used in order to fit data emerging from participants’ discussions into the pre-existing SPIKES Protocol frame and guide. Given that the goal of the study was to collect the views of ART professionals concerning the six steps of the SPIKES Protocol, the six steps represented an overarching frame for the analysis. In order to explore the explicit views of ART professionals about the applicability of the SPIKES Protocol, themes were deduced at a semantic level without including the underlying ideas and assumptions of professionals. Two researchers reviewed, coded and analyzed transcripts independently to reduce any bias. One of the two researchers was a facilitator of the focus group and the other one was an independent coder and an expert in communication. For reliability, comparisons between analyses were made. Discrepancies were solved through discussion with a senior researcher. The transcript was read several times by the two researchers in order to familiarize with data. Initial descriptive categories were formed and aggregated in themes. Thereafter, quotes explaining categories and themes were compared and themes were re-arranged accordingly. Themes were grouped in macro-themes and a hierarchical tree, subthemes and quotes were used to synthetize data. During this process, the two researchers constantly compared findings to align the analytic procedure and agree on developing themes. At the end of this process, participants were contacted by e-mail and were invited to provide feedback about the results obtained and to check the validity of the results.

## Results

The results of the discussion are presented in Table 3.

**Table 3:** Synthesis of the specificities in the ART context for each step of the SPIKES Protocol

DEFINITION OF BAD NEWS [38]	SPECIFITIES OF ART CONTEXT
“Any information which adversely and seriously affects the patient’s view of his or her future”	→ reiteration of bad news → the “patient” is a couple → there is not a disease to cure →existential failure for the couple → professional failure for the clinicians (“no enemy, no healing”)
SIX STEPS [22, 23]	SPECIFITIES OF ART CONTEXT
Setting up	→ communication by telephone → communication as a two- step process
Patient Perception	→ difficulty in managing the lack of information and/or the misinformation
Invitation	→ difficulty in balancing the couple desires to know with the clinicians’ need to be clear → besides what the couple wants to know, establishing what the patients have to know
Knowledge	→ joining the medical aspects of communication with the psycho-social ones → allowing patients to feel active and reduce their sense of powerlessness
Emotions	→ anger as the most difficult reaction to manage
Strategy and Summary	→ giving back to the couple a new meaning of the ART experience and an opportunity to grow

### Definition of bad news

Starting from Buckman’s definition of bad news – “Any information which adversely and seriously affects the patient’s view of his or her future.” [38] – participants highlighted that in the assisted reproductive medicine context there is a reiteration of bad news: infertility diagnosis, repeated treatment failures, the clinical ineffectiveness of medical treatments. Even the fact of seeking help from an ART or the presentation of menarche can be bad news. Unlike the oncological context, in assisted reproductive medicine the majority of the couples arrive knowing that there is a problem, and clinicians give the couple hope, a possible solution.

*“…it is an uninterrupted series of bad news…like something that starts rolling, and in the meanwhile even the period confirms the bad news” (Female, gynecologist)*

*“A cycle fails and after numerous cycles, the whole therapy fails” (Male, gynecologist)*

*“The first impact for the couple is not so negative: at the beginning we do not communicate anything negative, couples come with great hope and we offer therapies, the possibility to became pregnant…the bad news is when we have to stop the treatment (Male, gynecologist)”*

*“The shift from first to second level techniques for the patient is bad news, because you think ‘what difference will it make if in vitro fertilization is used as an alternative?’” (Female, ART patient)*

An element of complexity in assisted reproductive medicine seems to be that the “patient” is a couple and clinicians have to manage two perspectives that may conflict:

*“We need to remember that we are speaking to a couple and not a single patient…the communication could have a different effect: we are speaking to two persons who are receiving the same news, but who could react in completely different ways” (Female, gynecologist)*

Another specificity of the ART context, is that there is no disease to cure and the aim of the treatment is not to heal:

*“Who is the enemy? We do not work against a disease, but against a symptom. The object of our work is not lung cancer or pancreatic cancer; our object is the fact that the couple cannot reproduce” (Male, gynecologist)*

Participants spoke about bad news in ART as a failure, with the term “failure” having two main meanings:

1. *the existential failure for the couple*: participants highlighted how the inability to conceive are for the couple a failure of a whole life project with consequences on personal self-esteem, a sense of shame toward the family’s expectations and the perception of social stigmatization.

*“Femininity is always represented with a big womb…so there is the crushing of self-esteem, an awareness that the couple will never realize that project and that there is a biological clock that is expiring” (Male, gynecologist)*

*“It is a failure of an emotional project, of something that goes beyond illness and that deals with existence” (Female, obstetrician)*

*“After such a big, physical, emotional, economic investment, even time consuming, seeing everything collapse …it is difficult to get back up again” (Female, ART patient)*

Considering this existential meaning of the failure, participants perceived the impossibility to have a child as a real bereavement for couples. In their perception, the ART context was similar to the context of end of life care, where the concept of death needs to be elaborated and the grief for losing someone – in the ART context for losing a part of the self - needs to be processed.

*“You have to prepare couples to the idea that they might not be able to have children…Our work is similar to the work of an oncologist: I’ve seen death in the ART context, it is the death of women, of the couple, of self-esteem, of self-image…it is a different type of death, but still similar” (Male, gynecologist)*

2) *the professional failure for the clinicians*: participants highlighted how disappointment and frustration are not only for the couple, but also for the healthcare staff, who experience blame for their failure and the low rate of treatment success.

*“The first bad news is to need ART treatment, and the second is that ART treatments have no sure results” (Female, gynecologist)*

*“It seems that we need to excuse ourselves with patients for the fact of having to communicate that there is a 20% possibility of success. I do not think this happens in oncology” (Female, gynecologist)*

*“I was wondering if the professional failure in ART context could be considered peculiar due to the fact that there is not an ‘enemy’, the aim of the clinician is not the healing” (Male, health communication expert)*

Participants reported that on the one hand clinicians feel the power of helping the couple generate life, but also face the impotence of repeated failed treatments.

*“Just as in oncology, where the doctor does everything possible to help the patient live, in assisted reproductive medicine the doctor also helps the couple generate life, and when these possibilities are questioned, there is a big failure experience” (Female, psychologist)*

### Tracking the six-step SPIKES protocol

#### Step 1: Setting up the interview

Participants discussed the importance of setting up the interview when disclosing bad news. They highlighted that a careful set up of the interview is a good approach, that may be difficult to execute in every day practice.

*“We clearly adhere to the Buckman model; setting up the interview is fundamental: you need a place where to speak, you need time, you need to be prepared and well informed” (Male, gynecologist)*

Participants when discussing the first step debated two main specificities of the ART context:

1. *Communication by telephone*: most of the time the communication in assisted reproductive medicine is delivered by phone, including the communication of bad news such as a negative blood test or the failure of the gamete fecundation. Participants expressed their discomfort in managing such types of conversations and highlighted the need to be prepared and trained.

*“An everyday occurrence for me is to communicate a negative beta hCG and the consequent failure of the treatment…for me it is a big discomfort and I feel a sense of guilt… relationship for me is essential, if I do not see the patient, I am not able to understand the feedback, I can only interpret the pauses, the silences, but I do not understand the feelings” (Female, obstetrician)*

*“For what concerns phone consultations, clear instructions are necessary… we need to identify who is authorized to answer the telephone and be trained to communicate bad news” (Female, gynecologist)*

2) *Communication as a two-step process*: in some cases, such as when communicating the failure of the gamete fecundation, communication is given in two steps. The biologist who calls the patients usually manages the first communication; then, the doctor discusses in depth with the couple.

*“It is a two-step process, always: when the patient calls, the bad news has to be delivered, but it is difficult in that moment to have the time to face the matter in depth…but once the communication has been given, there is always a following face-to-face appointment with the doctor” (Male, gynecologist)*

*“In our Center for a certain period only the physicians gave the bad news, but we noticed that patients were suffering because they did not receive the biological explanation of what had happened…now biologists have restarted to call the patients and we observed a positive feedback from them” (Female, biologist)*

#### Step 2: Patient perceptions

Participants agreed with the importance of assessing what the couple already knows and has understood, as underlined in the SPIKES Protocol. However, they pointed out the difficulty in the ART context in managing patients’ lack of information and/or misinformation. Patients present with lofty (even unrealistic) expectations of treatment, often based on information obtained from the internet or other media sources.

*“It is plausible that some emotive mechanisms impede the comprehension of reality” (Female, psychologist)*

*“There is great misinformation. Couples generally do not know that the treatment has a low possibility of success. They do not know that at 45 years of age there are no possibilities of success” (Female, ART patient)*

*“What makes everything difficult is the lack of knowledge of the basic physiological aspects of reproduction: when you offer an ART treatment, couples think that success is automatic” (Male, gynecologist)*

#### Step 3: Invitation

Participants agreed that asking patients what they want to know is the most difficult step to apply in ART consultations, since it is difficult to balance what couples desire to know (or what they do not want to hear) with the clinicians’ need to be comprehensive. The exploration of couples’ expectations at the consultation allows an understanding of what couples want to know.

*“How can we apply Step 3? I agree with the approach of ‘Ask before talking’, understanding what couples’ expectations are, but defining with patients what I can explain and what I cannot is very hard” (Male, gynecologist)*

*“While with oncologic patients you can accept that they do not want to know about the probability of death, the ART context is different because anything that goes wrong, and was not explicit before, comes back in an explanation request from patients, who sometimes are also rancorous” (Female, gynecologist)*

Participants agreed that besides the matter concerning what couples want to know, it is fundamental for clinicians to establish what patients have to know, in order to share results and not foster unrealistic hopes.

*“Unlike oncology, in ART there is some information that patients need to know, they need to have awareness of the clinical data that is inalienable” (Male, expert in health communication)*

*“The reality check is the first point, it is as prerequisite to proceed” (Male, gynecologist)*

*“The uncertainty of the treatment, in my opinion, has to be present from the very outset. It is the unsaid that patients do not want to deal with” (Female, psychologist)*

*“At a certain point, you need to put a full stop: maybe patients do not want to know in the beginning, but then you have to communicate that the situation is critical” (Female, gynecologist)*

*“It is important to know that the results are not certain, it is an important point to start from, and of course hoping that you will obtain the pregnancy…undoubtedly the uncertainty is bad news, but it is a certain news that allows you to construct a psychological path…it is important for a patient to not live with too much illusions” (Female, ART patient)*

#### Step 4: Knowledge

Participants discussed the need in ART consultations to join the clinical aspects of communication, such as the biomedical contents of the ART visit with those psychosocial aspects that patients bring to the visit, such as their concerns and their personal history.

*“I rarely use the term ‘techniques,’ I prefer to talk about a ‘path’” (Male, gynecologist)*

*“It is a way to consider the reproductive inability not only from a biomedical point of view, but also at a psychosocial level: ART is not only percentages, failures or successes, embryos, numbers…it is also a matter of considering the history of the couple and of the single patient, their feelings, their desires and expectations” (Female, obstetrician)*

Several participants highlighted how some clinical information can have a psychosocial meaning for the patient and how it is important to allow patients to feel actively involved and reduce their sense of powerlessness.

*“Resting after the embryo transfer has no sense from a clinical point of view, but for the patient it could be useful to lie down, not go to work…as if it were possible to control an event on which there is no form of control whatsoever” (Female, gynecologist)*

*“An oncologic patient usually accepts to entrust the management of his/her disease to someone else…for what concerns fertility, a doctor should not be necessary, so with our therapies we are depriving couples of their autonomy” (Male, gynecologist)*

#### Step 5: Emotions

Participants agreed on the importance of being able to predict and address patients’ emotional reactions. They found great usefulness in Buckman’s suggestion to offer a tissue to a crying patient, which they saw as a sign of great respect. They found that anger is the most difficult reaction to manage, with the risk of reacting with anger as well, and they discussed some possible strategies to address patients’ emotions.

*“If they cry, at least I can offer tissues…the problem is when they do not cry, because if the patient gets angry, tissues are useless” (Female, gynecologist)*

*“We usually point out with a code in the medical chart the couples that, in our opinion, could have a strong emotional reaction” (Male, gynecologist)*

*“It is important not to consider anger as a personal attack against the doctor; you have to show them that you can understand that they are angry and you are sorry...if you react, you argue” (Female, gynecologist)*

*“…it helps to name all the anger, disillusion, discomfort…” (Male, gynecologist)*

*“The immense delusion of the failure of the first attempt…many patients fall in depression…the fall is physiological, clinicians should help patients to stand up again, in order to carry on with the treatments, when it is possible” (Female, ART patient)*

#### Step 6: Strategy and summary

Participants agreed that it was fundamental to wrap up the conversation by helping couples in elaborating their personal meaning of the clinical pathway they experienced. Participants highlighted the importance to move beyond the delivery of the bad news by giving back to the couple a new meaning of the ART experience and an opportunity to grow, in order to help them accept the situation and consider other possibilities (i.e. adoption, heterologous donation, childless).

*“It is crucial to retrace with the couple all the steps carried out, what we chose together, understanding how they feel, giving them a new interpretation, listening to the couple and offering an alternative” (Female, obstetrician)*

*“What can we give back to the couple, apart from the bad news? We have to value their efforts and help them make a different choice” (Male, gynecologist)*

*“Two years after the ART failure, a couple came to me with their two adopted children to introduce them to me…I understood that maybe I failed as an ART professional but not as a doctor” (Male, gynecologist)*

*“I think it is important to give value to the role of patients’ associations, that, besides clinicians, could help patients to accept other perspectives of life, adoption or even childlessness” (Female, ART patient)*

## Discussion

The present study aimed to explore, with a focus group of experts, if the SPIKES Protocol for breaking bad news [22, 23] can be applied to the ART context. ART experts who took part in the focus group generally agreed on the utility and adaptability of the SPIKES Protocol, finding it practical and easy to understand. During the discussion, participants found some similarities with the oncologic context, such as the theme of bereavement that the patient needs to elaborate or the clinicians’ sense of omnipotence/impotence. Domar et al. [9] underlined the parallelism with oncology, finding that the psychological impact of infertility had similarities to breast cancer and other serious medical conditions in terms of the symptoms and their intensity. Nonetheless, participants in the present study found some peculiarities of the ART context that need to be considered. In particular, the definition of bad news was considered more controversial than in oncology. Participants underlined some specific aspects for ART context that have been already pointed out by several authors [10, 19, 39, 40], such as the reiteration of bad news, the patient as a couple and the fact that infertility is seen as an existential failure. Unlike other medical contexts, where the bad news often deals with the patient’s survival, in the ART context the bad news is the denial of the existential possibility to conceive. This type of loss could be considered of low importance from the biomedical point of view, but could be a catastrophic from the patients’ perspective [3–9]. Communicating to patients that they were not able to conceive could have the same emotional burden as oncological bad news [9], ART clinicians are concerned with how best to communicate this information. Overall, participants in the present study found that the SPIKES Protocol may be a good guideline, which should however be adapted to ART. Also, the fact that in ART the patient is the couple, with two different individuals, needs to be taken into account since it adds an aspect of complexity.

Tracking step by step the SPIKES Protocol, participants highlighted some specificities of the ART context that need to be addressed. As far as the setting (Step 1), participants pointed out the discomfort of delivering bad news by phone, highlighting the need for the professionals who are prepared to manage the calls. The practice of delivering ART results by phone is common in assisted reproductive medicine, as also reported by Groh & Wagner [41]. In fact, they found that 96% of the patients interviewed received the ART results over the phone and the majority of them received the news unexpectedly and when alone. These findings suggest that couples should give preferences to when and how they are provided results. Undoubtedly, the communication of negative test results by phone is particularly challenging due to the lack of visual cues, and training in telephone medicine [42]. The issue of communicating bad news as a two-step process, discussed in the focus group as a best practice, seems to find confirmation in patients’ preferences explored by Groh & Wagner [41], who found that patients would have appreciated a follow-up call from the doctor or nurse to reduce the feelings of personal failure and abandonment.

The discussion of Steps 2 and 3 (assessing patients’ perception and what they want to know) highlighted the difficulties for clinicians to balance patients’ hopes and desires with the reality. The worries of managing patients’ misinformation and unrealistic expectations is common among clinicians, in an era in which the availability of medical information on the internet has changed the dynamics of the doctor-patient relationship [43]. This aspect needs to be further studied in order to support ART clinicians in managing the information that patients seek out.

For what concerns knowledge and information giving (Step 4), participants emphasized a need within all branches of medicine in the last decades: to consider not only the biomedical aspects, but also the psychosocial ones, according to a patient centered perspective. This point seems very important, since patient centered care is a component of high quality fertility care [44, 45] and related to increased patient well-being [46].

Discussing Step 5 (addressing patients’ emotions), participants highlighted a common concern for clinicians [47]: the unpredictability of patients’ reactions when giving bad news. As also noted by Grill [10], participants reported that patients’ anger is the most difficult emotion to manage. Even if the ability to address patients’ emotions empathically could be linked to personal attitudes, different studies have shown that experiential training in communication skills is effective to improve clinicians’ self-confidence, sense of preparation and practice in managing patient’s emotional reactions [48–51]. Finally, when discussing the last step of the SPIKES Protocol - strategy and summary - participants expressed the idea that success in assisted reproductive medicine stands not only in achieving pregnancy, but also in helping couples adapt to the bad news and facilitate a positive resolution of the crisis, as also pointed out by Lalos [19].

The present study had some limitations: only a sample of Italian ART experts were involved; it would be useful to repeat it with other ART professionals. Moreover, only one patient was recruited in the focus group. Caution is also advised in generalizing these findings broadly. Another limitation is that the group discussion may have been biased towards professionals with the most interest in communication issues, since some of them were recruited during a meeting on medical communication. Finally, future quantitative studies could be helpful to verify if the SPIKES Protocol is applicable and effective.

Despite its limitations, the study offered an opportunity to systematize suggestions and advice already existing in literature on how to deliver bad news in the infertility context, using the SPIKES Protocol as a model. These results seem consistent with the general (and few) recommendations offered by ART literature [10, 19]. They also highlight the importance of powering up a methodic process of discussion starting from the clinical practice of professionals involved in ART care in order to define a specific protocol, as other medical contexts such as oncology and end of life care have already done. The SPIKES Protocol has the benefit of being a practical and teachable method. Future studies should verify if a shared protocol, like SPIKES, could enhance ART clinicians’ confidence and preparation in engaging in difficult conversations with patients [48–51]. Future studies should also investigate the ART patients’ preferences for bad news delivery, in order to verify if a protocol like SPIKES could meet their needs. Moreover, future research should investigate if the use of a protocol for delivering bad news in the ART context could influence patients’ reactions, such as the decision to interrupt the treatment or the acceptance of a negative prognosis.

Working with a clear protocol on how to deliver bad news seems to have a beneficial effect on clinicians that experience less stress than those working without a protocol [47]. However, guidelines and protocols aimed to help manage difficult conversations can be considered only as a support since healthcare conversations are unique, and difficult conversations will continue to be difficult because of their nature [52]. Since the knowledge of bad news protocols alone is not enough, many authors [53–55] have highlighted the importance of offering experiential learning and practice opportunities.

## Conclusion

Overall the SPIKES Protocol seemed to be suitable for the ART context, especially for the practical suggestions to manage the conversation in its different steps. The issues that seem more challenging for the ART context are linked to the definition of bad news and to the management of the patients’ misinformation. The proposal of a shared protocol on how to give bad news tailored for the ART context could be the starting point for both experiential training and experimental studies on its efficacy.

## Abbreviations

ART: Assisted Reproductive Technology
SPIKES: Setting, Patient perception, Invitation, Knowledge, Emotions, Strategy and Summery
